# Characterization of the complete mitochondrial genome data of Asiatic Toad, *Bufo gargarizans* (Cantor, 1842) (Neobatrachia: Bufonidae) from Mianyang, China

**DOI:** 10.1016/j.dib.2025.112213

**Published:** 2025-10-30

**Authors:** Siqi Shan, Yi Qing, Simin Chen, Yaqing Liao, Dongmei Zhao, Lichun Jiang

**Affiliations:** a Key Laboratory for Molecular Biology and Biopharmaceutics, School of Biological and Pharmaceutical Sciences, Mianyang Normal University, Mianyang, Sichuan 621000, PR China; b Ecological Security and Protection Key Laboratory of Sichuan Province, Mianyang Normal University, Mianyang, Sichuan 621000, PR China

**Keywords:** *Bufo gargarizans*, Mitochondrial genome, Sequence analysis, Phylogeny

## Abstract

A common amphibian in East Asia, the *Bufo gargarizans* is significant for research on ecology, conservation biology, and evolution. Thus, the *B. gargarizans* mitochondrial genome was sequenced and subjected to a methodical analysis. The sequence was 17,431 base pairs long, with 13 protein-coding genes (PCG), 22 transfer RNA (tRNA) genes, 2 ribosomal RNA (rRNA) genes, and a D-loop control region. The nucleotide base composition of the mitochondrial genome was skewed toward AT content (57.17%), including adenine (28.80%), thymine (28.37%), cytosine (27.40%), and guanine (15.43%). For phylogenetic analysis, Bayesian inference (BI) techniques were used to build a phylogenetic tree from the mitochondrial genomes of 27 other species and the *B. gargarizans*. The findings demonstrated that species of the Bufonidae family formed a monophyletic group with the *B. gargarizans*. The mitochondrial genome of *B. gargarizans* will serve as an invaluable asset for future investigations into the evolution, taxonomy, and genetic preservation strategies of this species. Mitochondria genomic data can be found in GenBank under accession number PV083742.

Specifications Table

**Table:** 

Subject	Biosciences/Genomics
Specific subject area	Mitochondrionics
Data format	Raw, Analyzed
Type of data	Table, Figure
Data collection	Genomic DNA was extracted according to the traditional phenol-chloroform extraction method; Sequencing: Sequenced using Sanger sequencing; Mapping the whole gene structure of circular mitochondria using Proksee; Phylogenetic analysis: Phylogenetic tree extrapolation using BI
Data source location	Province/City/Town: Sichuan, Mianyang, Fucheng District Country: China Latitude and Longitude : 31°28′ 59.99″N, 104°37′ 0″E Sample Storage Facility: Genomic DNA was deposited in the Ecological Security and Protection Key Laboratory of Sichuan Province.
Data accessibility	Repository name: NCBI GenBank Data identification number: PV083742 Direct URL to data: https://www.ncbi.nlm.nih.gov/nuccore/PV083742/ Repository name: NCBI BioProject Data identification number: PRJNA1235100 Direct URL to data: https://www.ncbi.nlm.nih.gov/bioproject/PRJNA1235100 Repository name: NCBI BioSample Data identification number: SAMN47312921 Direct URL to data: https://www.ncbi.nlm.nih.gov/biosample/3609905 Repository name: NCBI SRA Data identification number: SRR32662259 Direct URL to data: https://www.ncbi.nlm.nih.gov/sra/?term=SRR32662259 Repository name: Zenodo data Data identification number: 10.5281/zenodo.15166860 Direct URL to data: https://zenodo.org/records/15166860

## 1 Value of the Data

• Analysis of the mitochondrial genome sequence allows us to construct a phylogenetic tree between the *Bufo gargarizans* and other species, revealing its evolutionary position among amphibians.
• Mitochondrial genome analysis can be used to speculate on past changes in the distribution range of the *Bufo gargarizans* and to explore the ecological niche differentiation and its response mechanism to environmental changes, which will help predict the impact of future climate change on its survival.
• Studying whether specific genes or genomic regions have experienced natural selection pressures can help us understand how *Bufo gargarizans* have adapted to different environmental conditions, such as low oxygen environments in plateaus.

## 2 Background

According to taxonomy, the *Bufo gargarizans* is one of the most species-rich amphibian families, belonging to the order Anura, family Bufonidae, and genus *Bufo*. The species can live in a wide range of habitats, such as meadows, farmland, and the borders of forests. It is found throughout most of China and its neighboring countries [1]. The *B. gargarizans* also plays an important role in traditional oriental medicine [2]. Because of its high mutation rate, low recombination, and matrilineal inheritance characteristics, the mitochondrial genome-the genetic material found in mitochondria-has been used extensively as a potent molecular marker for species identification, phylogenetic inference, population structure, genetic diversity, and dynamics studies [[3], [4]]. The *Bufo gargarizans* mitochondrial genome can be examined to learn more about the species' genetic diversity, population migration patterns, and environmental adaptability [5]. This study's phylogenetic tree and taxonomic status were established by utilizing and analyzing the mitochondrial genome's entire sequence, which offers an experimental foundation for the species' classification and conservation.

## 3 Data Description

**Fig. 1:**
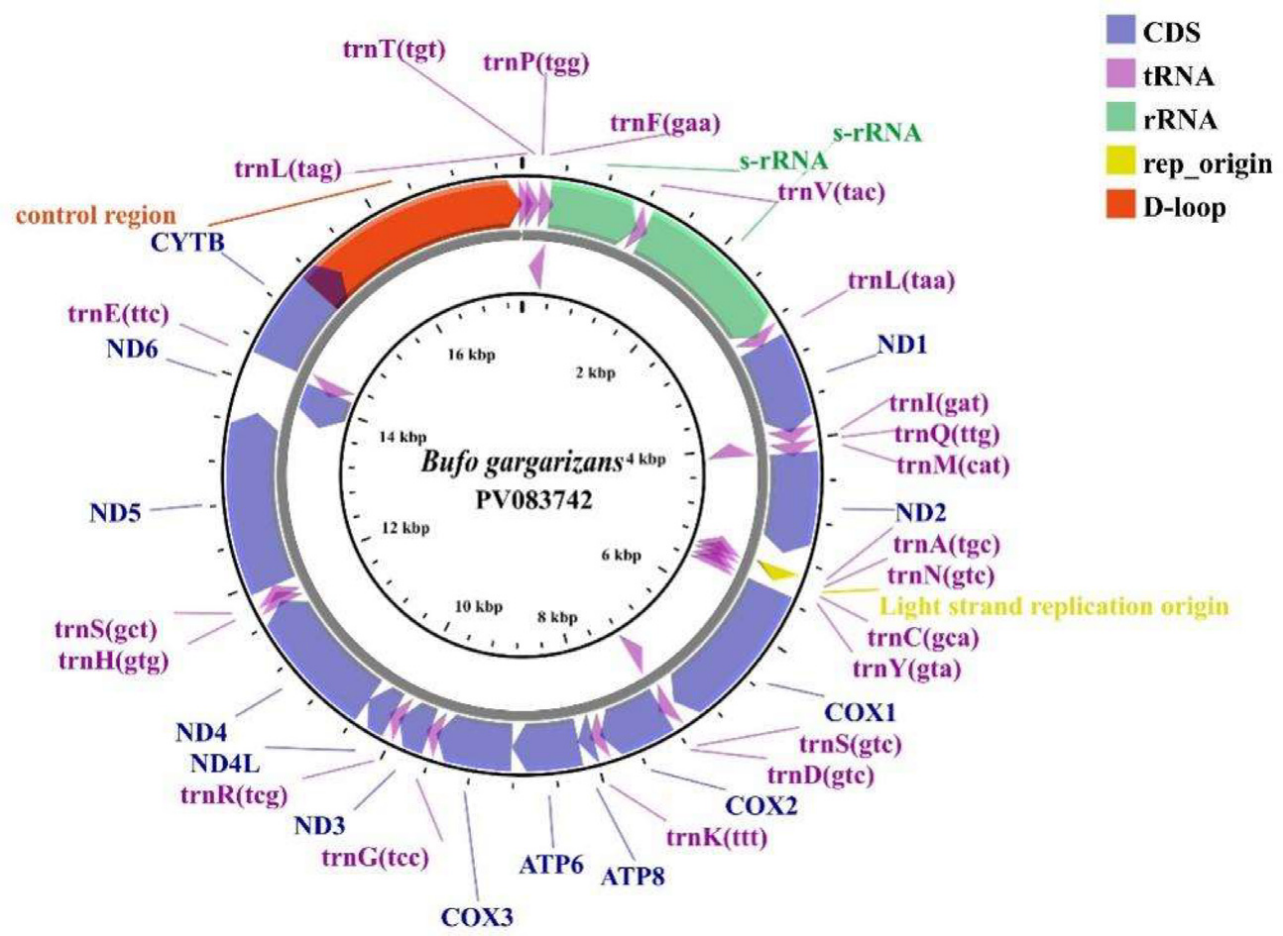
Complete mitochondrial genome organization and gene arrangement of *Bufo gargarizans*. Genes encoded on H- and l-strands with inverse arrow directions were shown outside and inside the circle, respectively.

The mitochondrial genome of *B. gargarizans* (GenBank PV083742) spans 174,531 bp in length, comprising 13 protein-coding genes, 2 ribosomal RNA genes (12S rRNA and 16S rRNA), 22 transfer RNA genes, and a non-coding regulatory region designated as the -loop (Fig. 1 and Table 1). Adenine (A) makes up a larger percentage of the nucleotide base composition of the mitotic genome (28.80 %), followed by thymine (T) at 28.37 %, cytosine (C) at 27.40 %, and guanine (G) at 15.43 %. As is typical of other amphibians, the percentage was low with the exception of guanine (G), while the other bases stayed relatively constant [6]. The *A* + *T* content, which is the sum of the adenine and thymine, makes up 57.17 % of the nucleotide composition.

**Table 1:** Annotation of the mitochondrial genome of *Bufo gargarizans.*

Gene	Direction	Position		Size (bp)	IGS (bp)	Codon	
		From	To			Start	Stop
tRNA-Leu	H	1	72	72	0		
tRNA-Thr	H	73	144	72	−1		
tRNA-Pro	L	144	212	69	−1		
tRNA-Phe	H	212	279	68	0		
12S ribosomal RNA	H	280	1209	930	0		
tRNA-Val	H	1210	1280	71	1		
16S ribosomal RNA	H	1282	2885	1604	1		
tRNA-Leu	H	2887	2959	73	5		
ND1	H	2975	3920	946	10	ATT	T–
tRNA-Ile	H	3921	3991	71	−1		
tRNA-Gln	L	3991	4061	71	−1		
tRNA-Met	H	4061	4129	69	0		
ND2	H	4130	5162	1033	0	ATA	T–
tRNA-Trp	H	5163	5232	70	0		
tRNA-Ala	L	5233	5301	69	0		
tRNA-Asn	L	5302	5374	73	0		
rep_origin	H	5375	5403	29	−3		
tRNA-Cys	L	5401	5464	64	0		
tRNA-Tyr	L	5465	5534	70	4		
COI	H	5539	7080	1542	2	ATA	TAA
tRNA-Ser	L	7083	7153	71	1		
tRNA-Asp	H	7155	7223	69	1		
COII	H	7225	7912	688	0	ATG	T–
tRNA-Lys	H	7913	7984	72	1		
ATP8	H	7986	8150	165	−10	ATG	TAA
ATP6	H	8141	8824	684	−1	ATG	TAA
COIII	H	8824	9607	784	1	ATG	T–
tRNA-Gly	H	9608	9676	69	0		
ND3	H	9677	10,016	340	0	ATG	T–
tRNA-Arg	H	10,017	10,085	69	0		
ND4L	H	10,086	10,385	300	−7	ATG	TAA
ND4	H	10,379	11,743	1365	0	ATG	TAA
tRNA-His	H	11,744	11,812	69	0		
tRNA-Ser	H	11,813	11,879	67	39		
ND5	H	11,917	13,719	1083	−17	ATG	AGA
ND6	L	13,703	14,197	495	0	ATG	AGA
tRNA-Glu	L	14,198	14,265	68	4		
CYTB	H	14,270	15,415	1146	0	ATG	AGA
D-loop	H	15,416	17,431	2016	0		

The mitochondrial genome of *B. gargarizans* exhibits a strand-specific distribution of its 37 genes: the *ND6* gene and eight tRNA genes (including genes encoding *tRNA^Gln^, tRNA^Ala^, tRNA^Asn^, tRNA^Cys^, tRNA^Tyr^, tRNA^Ser^, tRNA^Glu^,* and *tRNA^Pro^*) are located in the light chain, and the remaining genes are located in the heavy chain. All of the protein-coding genes begin with an ATG codon, while the *ND1* gene begins with ATT, followed by the *ND2* and *COI* genes with *ATA. COI, ATP8, ATP6, ND3, ND4L*, and *ND4* have TAA termination codons, while *Cytb, ND5,* and *ND6* have AGA termination codons. The remaining genes have incomplete T-type termination codons.

The 12S and 16S rRNAs made up the *B. gargarizans* mitochondrial rRNAs. The 12S rRNA began at 280 bp, ended at 1209 bp, and was 930 bp long, while the 16S rRNA began at 1282 bp, ended at 2885 bp, and was 1604 bp long. The non-coding regions included the d-loop region and a few non-coding spacer sequences, and the d-loop region was localized between 15,416 bp and 17,431 bp and was 2016 bp in length. The d-loop region, which is 1872 bp long and located between 15,406 bp and 17,431 bp, is part of the non-coding region along with a few non-coding spacer sequences. The 38 bp distance between *tRNA^Ser (AGY)^* and *ND 5* is the longest of these spacer regions.

**Fig. 2:**
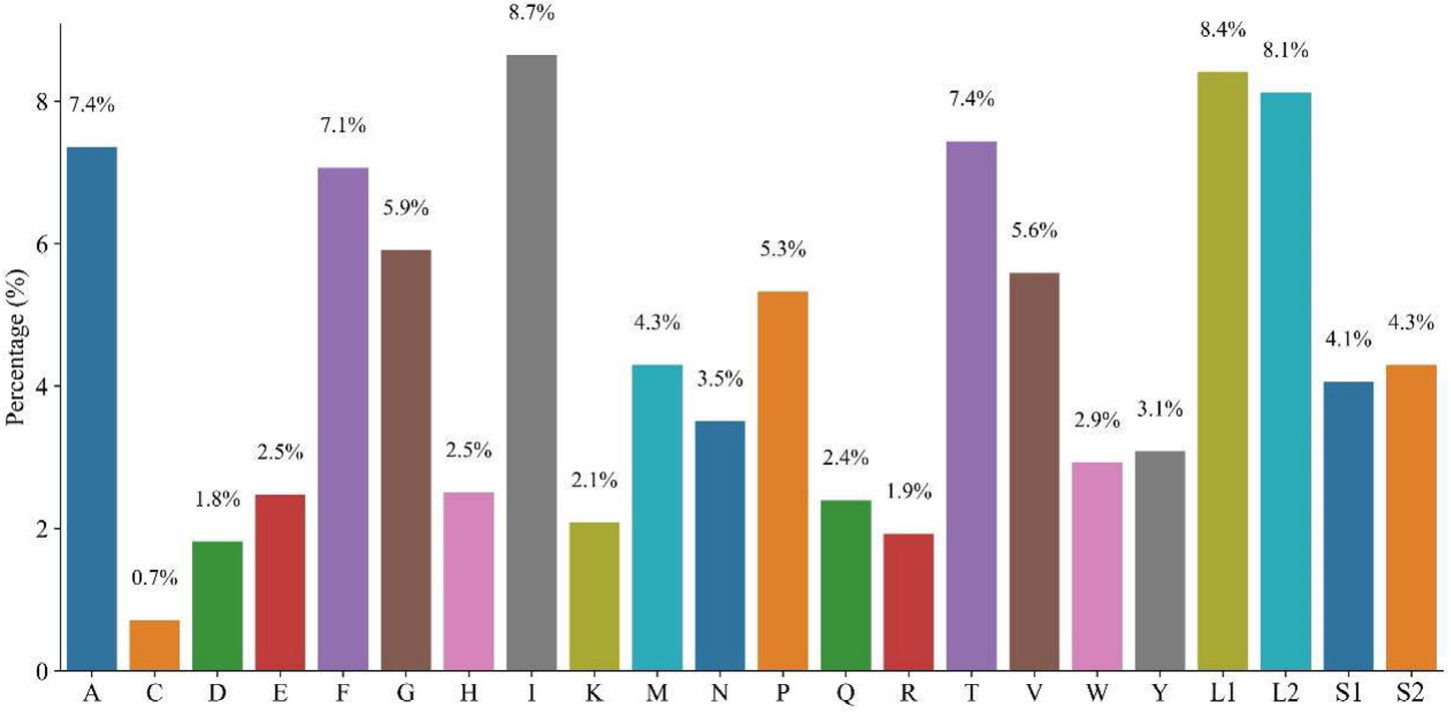
Proportional distribution of the composition of 22 standard amino acids in the proteome of *B. gargarizans*.

The amino acid content analysis (Fig. 2) revealed that the levels of L (leucine), I (isoleucine), A (alanine), S (serine), T (threonine), and F (phenylalanine) were all greater than 6 %. The presence of L was the most abundant amino acid, and its expression level differed significantly from that of the other amino acids, with L (leucine) and serine (S) classified as L1, L2 and S1, S2. Aspartic acid (D) and cysteine (C) were lower at 1.8 % and 0.7 %, respectively. This might have a direct connection to its oxidative sensitivity and the mitochondrial environment's restriction of disulfide bond formation. These results offer a database for researching the evolution of *B. gargarizans* proteins and their adaptation to evolutionary pressures through amino acid utilization.

**Fig. 3:**
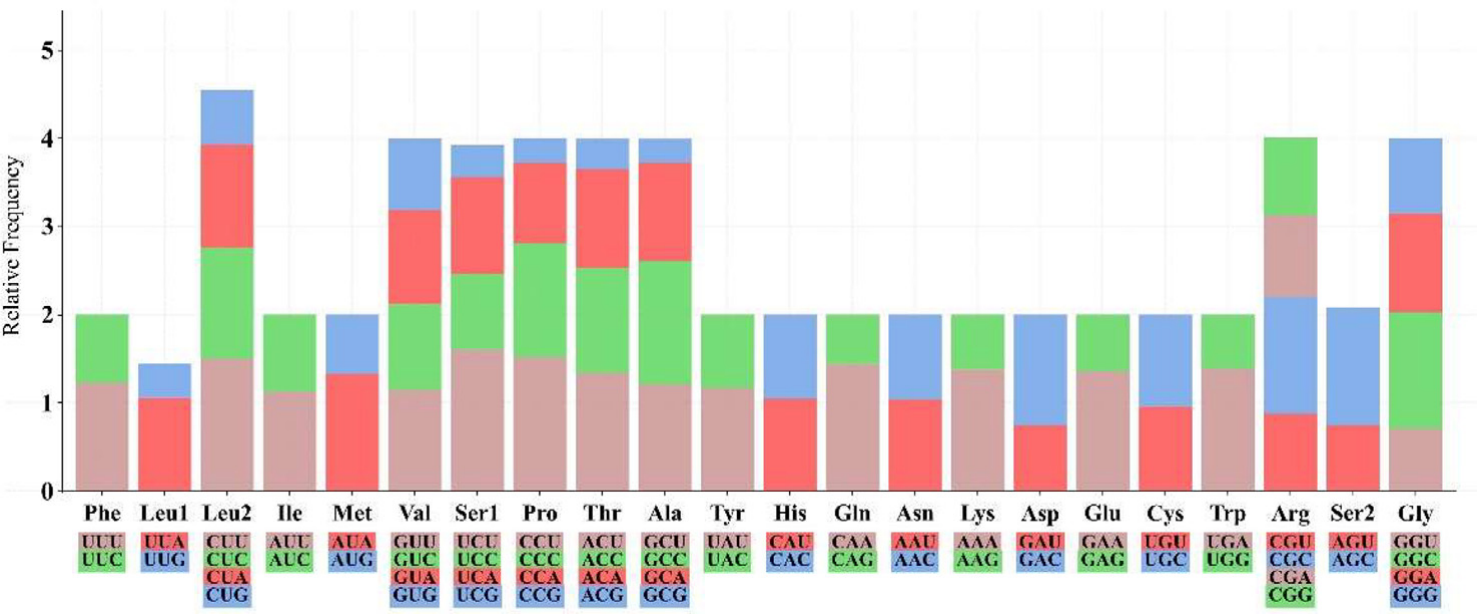
Relative Synonymous Codon Usage (RSCU) in *B. gargarizans*. Codon families are provided on the X axis and the RSCU values on the Y axis.

The results of the relative codon frequencies of the *B. gargarizans* are shown in Fig. 3 The RSCU values of all amino acid codons were counted, and it was found that the four codons that were used more frequently were UCU (Ser1), CCU (Pro), CAA (Gln), and UGA (Trp), and the codons that were used less frequently were GCG (Ala), CCG (Pro), UCG (Ser1), and ACG (Thr). The frequency of codon usage varies greatly among amino acids, with leucine (Leu) exhibiting the most typical bidirectional divergence. The relative frequency of codons belonging to the Leu2 class (such as UUG and CUG) is significantly higher than that of the Leu1 class, indicating that it may optimize translation efficiency by enriching high-frequency codons or be associated with adaptive matching of tRNA abundance.

**Fig. 4:**
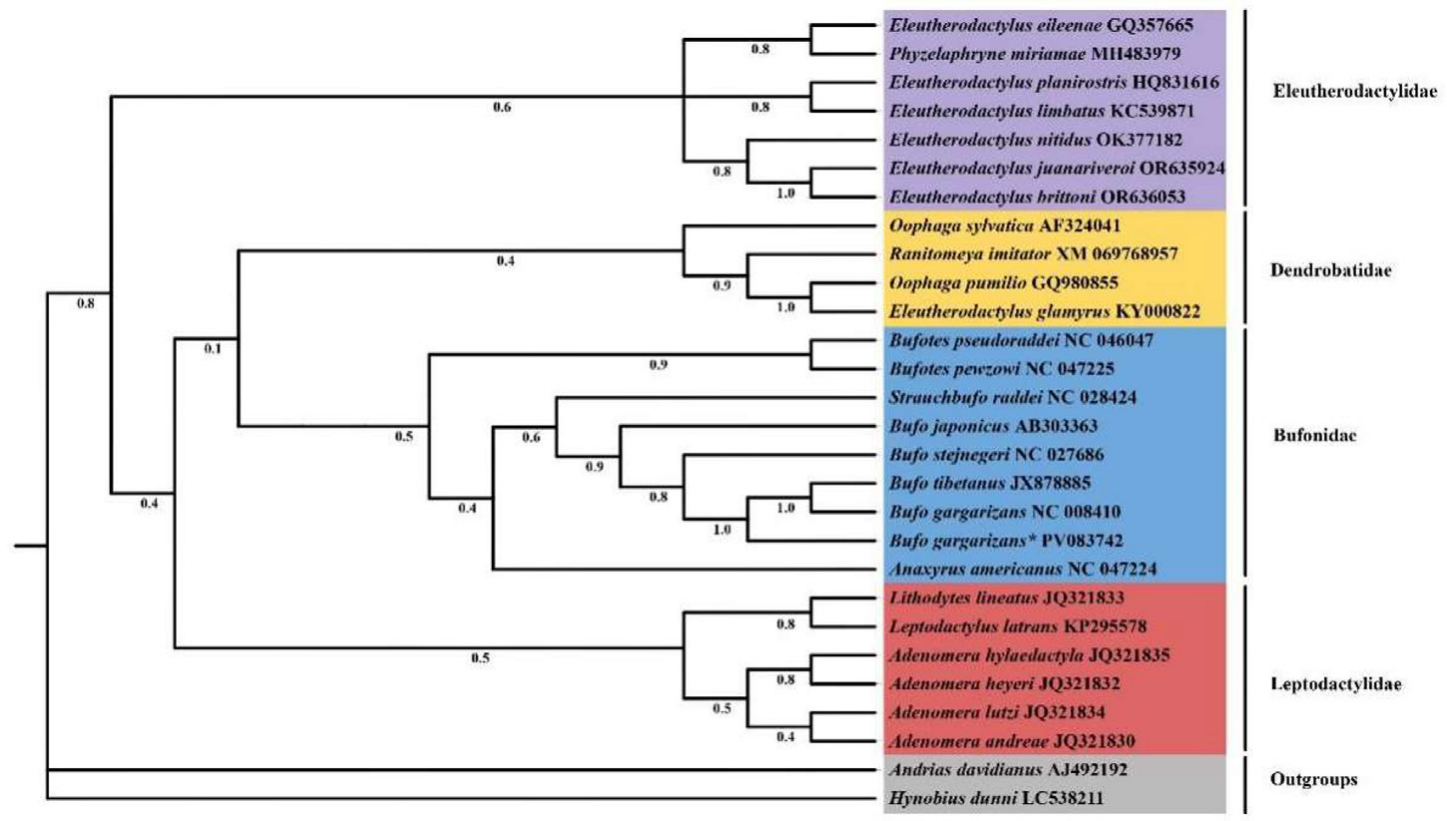
The phylogenetic tree inferred by the Bayesian inference (BI), phylogenetic relationships of B. gargarizans* and other 27 species based on 13 protein-coding genes (PCGs). *Hynobius dunni* and *Andrias davidianus* are used as outgroups. GenBank accession numbers and bootstrap values are shown on the tree. The asterisks indicate new sequences generated in this study.

Four amphibian families (Eleutherodactylidae, Dendrobatidae, Bufonidae, and Leptodactylidae) were divided into distinct branches based on the mitochondrial DNA genome sequences of 28 species, as illustrated in Fig. 4. Furthermore, two species (*Andrias davidianus* and *Hynobius dunni*) are regarded as classification outgroups. Based on the chosen evolutionary model, a phylogenetic tree was created using Bayesian inference (BI) [7]. According to the results, *B. tibetanus* and *B. gargarizans* clustered together to form a branch, indicating that they are closely related and form part of the family Bufonidae. *B. gargarizans* forms a monophyletic group with its congeners. The relationships within the families Dendrobatidae and Bufonidae, which form a sister group to the family Leptodactylidae and then cluster with the family Eleutherodactylidae, have high support values. These results contribute to the understanding of the evolutionary relationships and taxonomic status of the different species.

This study revealed that the *B. gargarizans* genome exhibits a dN/dS ratio (ω) below 1 (Table 2), indicating strong purifying selection acting on specific genes during evolutionary processes. This selective pressure ensures remarkable functional conservation of these genetic elements, consistent with the essential roles mitochondrial proteins play in core cellular functions including energy transduction and other fundamental biochemical processes. Such evolutionary constraints likely reflect the deleterious consequences of mutations compromising these vital functions. This molecular evolutionary pattern provides insights into *B. gargarizans'* exceptional ecological plasticity, potentially explaining its status as one of the most widely distributed amphibian species across diverse ecosystems.

**Table 2:** The Ka/Ks values among the *Bufo gargarizans* species.

	ATP6	ATP8	COX1	COX2	COX3	CYTB	ND1	ND2	ND3	ND4	ND4L	ND5	ND6
PV083742-VS- AB303363	0.0128	0.1177	0.0216	0.0393	0.0038	0.0113	0.0124	0.0381	0.0629	0.0418	0.0392	0.0322	0.0497
PV083742-VS- NC_028424	0.0221	0.0842	0.0055	0.0103	0.0129	0.0142	0.0195	0.0318	0.0278	0.0415	0.0309	0.0411	0.0727
PV083742-VS- NC_027686	0.0109	0.1236	0.0166	0.0456	0.0143	0.0254	0.0333	0.0278	0.0454	0.0498	0.0187	0.033	0.0725
PV083742-VS-NC_020048	0.0452	0.3887	0.0137	0.0081	0.0537	0.0316	0.0309	0.0865	0.001	0.0828	0.001	0.0474	0.0587

Note: PV083742, AB303363, NC_028424, NC_027686, and NC_020048 represent *Bufo gargarizans*(this study), *Bufo japonicus, Bufotes raddei, Bufo stejnegeri,* and *Bufo tibetanus*, respectively.

## 4 Experimental Design, Materials and Methods

### 4.1 Sampling, DNA extraction, and DNA sequencing

The samples of *B. gargarizans* in this experiment were taken from the population samples of Yongxing Town (Latitude: 31°28′ 59.99″N, Longitude: 104°37′ 0″E), Fucheng District, Mianyang City, Sichuan Province, Samples were collected using a sampling tool under sterile conditions and were immediately preserved in vials containing appropriate preservative solution, and subsequently stored in a refrigerator at −20 °C until use [8].

This experiment's DNA extraction technique primarily refers to the conventional phenol-chloroform extraction method for extracting genomic DNA [9], and the DNA sequence was amplified using conventional PCR technology. An agarose gel was prepared with a concentration of 1.0–2.0 % (the gel's concentration was suitably adjusted based on the length of the reaction product). Electrophoresis was used to identify the reaction products at the conclusion of the DNA amplification reaction; those that had a single destination band and clear brightness were forwarded straight to the business for sequencing [10].

### 4.2 Mitogenome assembly and annotation

Sanger sequencing technology [11] was utilized in a thorough examination of the *B. gargarizans* mitochondrial genomic data to obtain whole genome data with high accuracy. Table 1 displays the mitochondrial whole-gene structure characterization table. FastQC was used to evaluate the quality of the sequencing data produced from the *B. gargarizans* samples that were gathered [12]. The majority of the reads had high quality scores (Q30 > 85 %), according to the results, suggesting that the data was of good quality overall and appropriate for further analysis. Trimmomatic was used to eliminate low-quality read segments and splice sequences based on the FastQC results [13]. The mitochondrial genome was reconstructed de novo using NOVOPlasty v.4.3.1 [14] under default settings to generate candidate mitogenome sequences. The sequence assembled by NOVOPlasty was used as a reference for mapping using BWA-MEM [15]. The circular structure of the mitochondrial genome was graphically represented and analyzed using CGVIEW [16].

### 4.3 Phylogenetic analysis

To investigate the phylogenetic relationships of *B. gargarizans,* a Bayesian Inference (BI) approach was employed to construct and analyze a phylogeny using 37 genes (2 rRNA genes, 22 tRNA genes, and 13 protein-coding genes) from 28 related species, including 7 species of Eleutherodactylidae, 4 of Dendrobatidae, 9 of Bufonidae, 6 of Leptodactylidae, and 2 outgroups. Bayesian analysis was performed using MrBayes v3.2, with sequences from the same species concatenated using SequenceMatrix. The nucleotide dataset was modeled under the GTR + *G* + *I* substitution model. Four Monte Carlo Markov chains (MCMC) were initiated with random starting trees and run for 500,000 generations. Trees were sampled every 1000 generations, with the first 25 % of samples discarded as burn-in to ensure parameter convergence. Multiple sequence comparisons using MAFFT [17] and the creation of a phylogenetic tree verified the *B. gargarizans* unique position at the mitochondrial genome level and its relationship with other toad families.

## Limitations

Not applicable.

## Ethics Statement

The study adheres strictly to relevant ethical guidelines for scientific research and adheres to the principles of good faith, openness and transparency in data collection, analysis and reporting of results, respecting the right to informed consent and privacy of all participants.

## **Data Availability**

*Bufo gargarizans* mitochondrion, complete genome (Original data) (NCBI GenBank).

## CRediT author statement

**Siqi Shan:** Investigation, Collect samples, Analyze the data, Writing-Original draft; **Yi Qing:** Investigation, Collect samples, Conceive and designed the experiments; Contribute analysis tools; **Simin Chen:** Investigation; Collect samples; Contribute analysis tools; Organize tables and beautify pictures, Administration project; **Yaqing Liao:** Investigation, Analyze data, prepare reports and tables; **Dongmei Zhao**: Analyze data, Conceive and designed the experiments, Organize tables and beautify pictures; **Lichun Jiang:** Conceive and designed the experiments, Write the paper, Visualization, Supervision, Administration project, Funding acquisition.

## Data Availability

Bufo gargarizans (Original data)
